# Photoacoustic imaging of the human placental vasculature

**DOI:** 10.1002/jbio.201900167

**Published:** 2019-11-25

**Authors:** Efthymios Maneas, Rosalind Aughwane, Nam Huynh, Wenfeng Xia, Rehman Ansari, Mithun Kuniyil Ajith Singh, J. Ciaran Hutchinson, Neil J. Sebire, Owen J. Arthurs, Jan Deprest, Sebastien Ourselin, Paul C. Beard, Andrew Melbourne, Tom Vercauteren, Anna L. David, Adrien E. Desjardins

**Affiliations:** ^1^ Wellcome/EPSRC Centre for Interventional and Surgical Sciences, University College London London UK; ^2^ Department of Medical Physics and Biomedical Engineering University College London London UK; ^3^ Institute for Women's Health, University College London London UK; ^4^ School of Biomedical Engineering and Imaging Sciences, King's College London London UK; ^5^ Research and Business Development Division CYBERDYNE INC Rotterdam the Netherlands; ^6^ NIHR Great Ormond Street Institute of Child Health Biomedical Research Centre, University College London London UK; ^7^ Department of Histopathology Great Ormond Street Hospital for Children NHS Trust London UK; ^8^ Paediatric Radiology, Great Ormond Street Hospital for Children NHS Trust London UK; ^9^ Department of Obstetrics and Gynaecology University Hospitals Leuven Leuven Belgium

**Keywords:** fetal therapy, human placenta imaging, photoacoustic imaging, twin‐to‐twin‐transfusion syndrome

## Abstract

Minimally invasive fetal interventions require accurate imaging from inside the uterine cavity. Twin‐to‐twin transfusion syndrome (TTTS), a condition considered in this study, occurs from abnormal vascular anastomoses in the placenta that allow blood to flow unevenly between the fetuses. Currently, TTTS is treated fetoscopically by identifying the anastomosing vessels, and then performing laser photocoagulation. However, white light fetoscopy provides limited visibility of placental vasculature, which can lead to missed anastomoses or incomplete photocoagulation. Photoacoustic (PA) imaging is an alternative imaging method that provides contrast for hemoglobin, and in this study, two PA systems were used to visualize chorionic (fetal) superficial and subsurface vasculature in human placentas. The first system comprised an optical parametric oscillator for PA excitation and a 2D Fabry‐Pérot cavity ultrasound sensor; the second, light emitting diode arrays and a 1D clinical linear‐array ultrasound imaging probe. Volumetric photoacoustic images were acquired from ex vivo normal term and TTTS‐treated placentas. It was shown that superficial and subsurface branching blood vessels could be visualized to depths of approximately 7 mm, and that ablated tissue yielded negative image contrast. This study demonstrated the strong potential of PA imaging to guide minimally invasive fetal therapies.

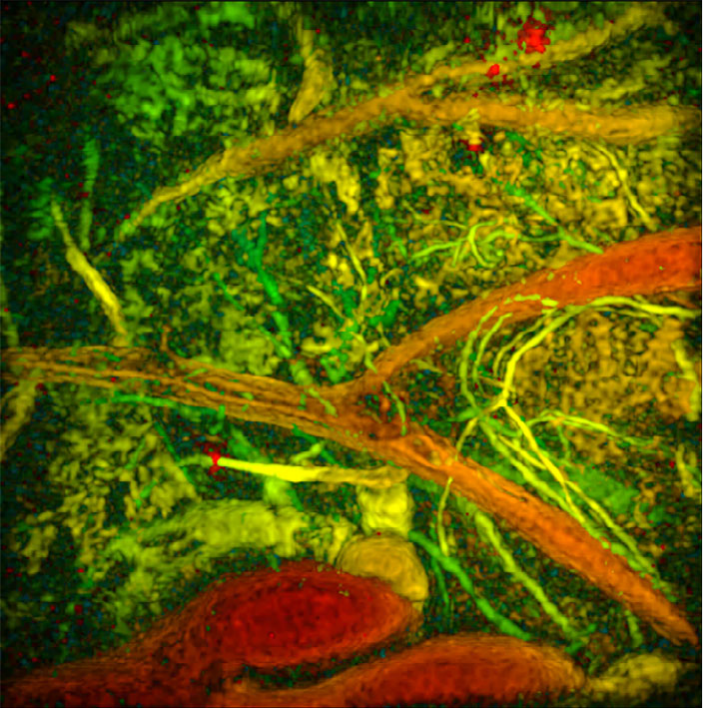

## Graphical abstract

Successful therapy of twin‐to‐twin transfusion (TTTS) syndrome requires accurate imaging to guide laser photocoagulation of the anastomosing placental vessels. Here, two volumetric photoacoustic imaging systems with optical (Fabry‐Pérot) and piezoelectric ultrasound receivers were used to image human ex vivo normal and TTTS‐treated placentas. With imaging depths of approximately 7 mm, vasculature and ablated regions were visualized. The strong potential of photoacoustic imaging to guide minimally invasive fetal therapies was demonstrated.

## 1 INTRODUCTION

The human placenta is the interface between the mother and the developing fetus, delivering the nutrients and oxygen vital for normal growth and development 1. In monochorionic twin pregnancies, conditions such as selective fetal growth restriction and twin‐to‐twin transfusion syndrome (TTTS) are associated with specific placental angioarchitectural patterns 2, 3. TTTS, a condition considered in this study, occurs from an imbalance of blood flow across inter‐twin vascular anastomoses in the placenta; it is associated with a high risk (80%–90%) of perinatal mortality if left untreated 4. Minimally‐invasive fetoscopic laser photocoagulation of the vascular anastomoses is used to treat TTTS 5, 6, 7. In current clinical practice, two imaging modalities are used during the intervention: B‐mode ultrasound (US) imaging to guide instruments from outside the mother into the uterus to the placenta, and white light fetoscopy within the uterus to identify vascular anastomoses on the chorionic (fetal) placental surface for photocoagulation. However, both of these modalities provide insufficient contrast to visualize small anastomosing vessels beneath the chorionic placental surface 8. Missed anastomoses or incomplete photocoagulation are associated with an increased risk of recurrent TTTS, intrauterine fetal death and twin anemia polycythaemia sequence 9, 10, 11, 12. With the procedural aim of occluding all anastomoses and thereby separating the twins' circulations, the “Solomon technique” involves photocoagulation of the entire vascular equator 12, 13. In one randomized control trial, complete coagulation of the entire vascular equator significantly reduced postoperative fetal morbidity, as compared with selective occlusion 13. However, residual anastomoses remained in around 20% of placentas; while this technique is very promising, there is room for refinement. Advancements in methods for imaging placental vasculature in vivo 14, 15 could lead to significant improvements in the treatment of TTTS.

Photoacoustic (PA) imaging is an emerging imaging modality that provides molecular contrast from the optical absorption of excitation light 16, 17, 18. With PA imaging, contrast from vasculature can be particularly prominent due to optical absorption by hemoglobin, as observed in many clinical contexts 19, 20, 21, 22, 23, 24, 25, 26, 27, 28. PA imaging has been used to measure in vivo placental oxygenation under conditions of maternal hypoxia and hyperoxygenation 29 and preeclampsia 30 in rats, and in pregnancies associated with hypertension and fetal growth restriction in mice 31 respectively. Previously, two‐dimensional (2D) PA images were acquired from ex vivo healthy term singleton human placentas with a clinical linear‐array US imaging probe and delivery of excitation light through an optical fiber adjacent to the placenta 32, 33. This data provided an initial demonstration that human chorionic placental vessels can be visualized with 2D PA imaging. In the present study, we performed three‐dimensional (3D) PA imaging of two ex vivo human placentas: a normal, term placenta and a placenta from an identical twin pregnancy complicated by TTTS and treated with photocoagulation in utero. The motivations for our investigations were twofold: first, to explore the potential of PA imaging of postpartum placentas for improving our understanding of TTTS and the effects of photocoagulation; second, to appreciate what information may be available from intraoperative PA imaging, with future probes that would be suitable for intrauterine imaging. The normal and TTTS‐treated placentas were first imaged postpartum using a reference Fabry‐Pérot (F‐P) based planar sensor PA imaging system, and subsequently with a portable dual‐modality PA/US imaging system based on a clinical linear‐array US imaging probe that was mechanically scanned to create 3D images.

## 2 MATERIALS AND METHODS

### 2.1 Human placentas

The placentas were obtained with written informed consent after caesarean section deliveries at University College London NHS Foundation Trust and Lister (East and North Hertfordshire NHS Trust) Hospitals. The Bloomsbury National Research Ethics Service Committee London approved the study (14/LO/0863). The umbilical cords were clamped immediately after delivery to preserve the blood inside the vessels. Once the placenta separates from the uterine myometrium after birth, the intervillous space into which maternal blood circulates under low pressure collapses. Clamping the umbilical cord at the birth of the baby preserves the fetal blood compartment inside the fetal vessels. Blood can leak out if structural damage occurs to the fetal vessels in the placenta during delivery. The normal, term placenta was delivered after an uncomplicated pregnancy at 38 weeks +6 days gestational age (GA). The patient who provided the identical twin placenta was successfully treated for TTTS at 16 weeks +4 days GA using fetoscopic laser ablation by complete coagulation of the entire vascular equator, and was then delivered by planned Caesarean section at 34 weeks GA. Imaging was performed in several locations on the chorionic surface within 1 to 4 hours of delivery.

### 2.2 Fabry‐Pérot‐based planar sensor PA imaging system

**Figure 1:**
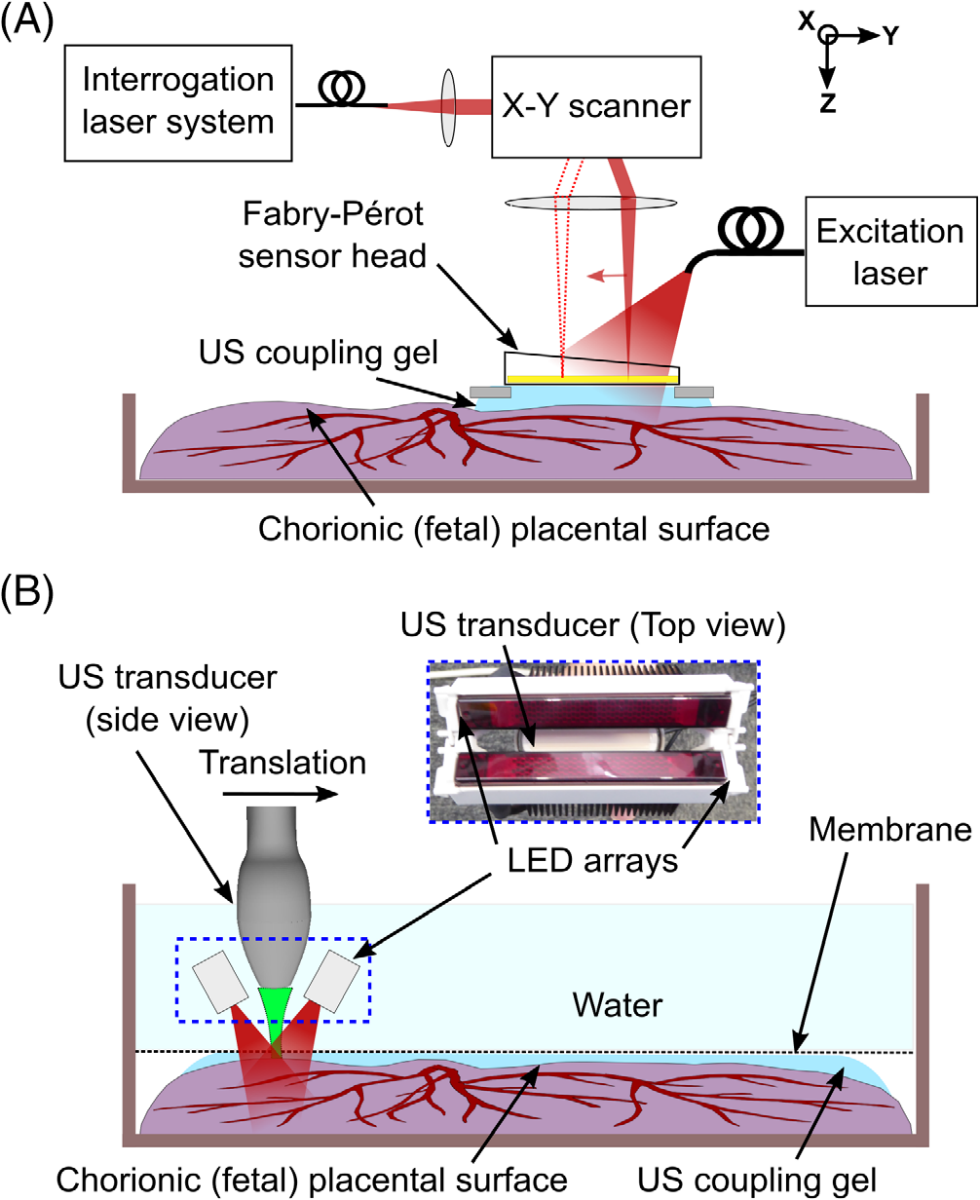
Photoacoustic imaging experimental setups. A, Fabry‐Pérot‐based planar sensor PA imaging system. The sensor head gently touched the placenta from the chorionic (fetal) surface, with ultrasound (US) gel placed in between the sensor and the tissue for acoustic coupling. B, Clinical linear‐array ultrasound probe‐based PA imaging system. The US imaging probe with light emitting diode (LED) arrays was translated across the placenta using a linear motorized stage. The placenta was coated with US gel, covered with a plastic membrane (cling film) and placed inside a water‐filled container. The plastic membrane minimized leakage of blood from the placenta to the water above it

The first PA imaging system used for placental imaging comprises an optical parametric oscillator (OPO) to deliver excitation light and a planar F‐P sensor for ultrasound reception (measured −3 dB acoustic bandwidth: 22 MHz 34; Figure 1A). The operating principles of this reference approach have previously been described in detail 34, 35, 36. The F‐P ultrasound sensor consists of a transparent polymer film spacer sandwiched between two mirrors. The thickness of this spacer is modulated by impinging ultrasound waves, which in turn modulate the optical reflectivity of the sensor. These modulations were measured using a continuous wave interrogation laser beam that was raster‐scanned across the surface of the F‐P sensor. The mirrors of the F‐P sensor were designed to be transparent at the wavelength of the excitation light pulses (Figure 1A). All images were obtained using an excitation wavelength of 760 nm and a fluence below 2.5 mJ/cm^2^. The excitation light wavelength was chosen to optimize the balance between contrast and penetration depth. For the normal term placenta, a fiber‐coupled Nd:YAG pumped OPO (Spitlight 600, InnoLas Laser GmbH, Krailling, Germany) operating at 30 Hz was used. For the TTTS‐treated placenta, a different OPO (InnoLas Laser GmbH, Krailling, Germany) operating at 200 Hz was used. The PA imaging system has a lateral field of view of 14 mm × 14 mm, a depth‐dependent spatial resolution in the range of 50 to 125 μm, and a typical tissue penetration depth of approximately 10 mm, depending on the excitation light wavelength.

Image reconstruction was performed using a frequency‐domain algorithm implemented with the “k‐wave” Matlab toolbox 37, using a sound speed of 1540 or 1560 m/s. This variation in the optimal speed of sound used for reconstruction may have originated from differences in the samples' temperatures. Post‐processing steps, including normalizing image intensity with depth, were previously described by Jathoul et al 35. The reconstructed images were displayed as depth color‐coded maximum intensity projections (MIPs) along different dimensions (*x*‐*y*: lateral dimensions; *z*: depth dimension). Although this display typically incurs some information loss, the full 3D volumetric datasets were saved and exported in DICOM format. Efforts were made to apply a minimum of pressure to position the sensor onto the placenta; ultrasound gel between the sensor and placenta was used for acoustic coupling.

### 2.3 Clinical linear‐array ultrasound probe‐based PA imaging system

The second PA imaging system used for placental imaging acquired co‐registered PA and B‐mode US images with a clinical linear‐array US imaging probe (AcousticX, CYBERDYNE INC., Tsukuba, Japan) 38, 39, 40. Excitation light for PA imaging was provided by two light emitting diode (LED) arrays (850 nm wavelength; 200 μJ nominal pulse energy per array; 4 kHz pulse repetition frequency) positioned on opposite sides of the imaging probe. The excitation light wavelength of 850 nm was chosen due to the availability of this LED array. The probe has a central frequency of 9 MHz, 128 elements with a 300 μm pitch and a nominal −6 dB acoustic bandwidth of 77% 40 (Figure 1B). This probe provides a PA spatial resolution of 0.22 ± 0.01 mm and 0.46 ± 0.06 mm across the axial and lateral dimension respectively, as characterized previously by Xia et al 40. 3D PA imaging was enabled with translation of the imaging probe with a linear motorized stage supplied with the system (OSMS20‐85[X], OptoSigma Corporation, California). The translated distance during each 3D volume acquisition was 10 mm. Concatenation of these 3D volumes yielded a field of view of 38 mm × 60 mm. Upsampling (3×) in the lateral dimension of the raw data and offline PA image reconstruction was performed using a frequency‐domain algorithm implemented with the “k‐wave” Matlab toolbox 37 and a sound speed of 1540 m/s. The placenta was initially placed in a plastic container, coated with ultrasound gel for acoustic coupling and covered with a plastic membrane (cling film). The container was then filled with water at room temperature for acoustic coupling, and for free translation of the US imaging probe.

### 2.4 Histology

A portion of the placenta treated for TTTS that included photocoagulated (scar) tissue resulting from the laser photocoagulation was dissected into a 15 mm × 15 mm full thickness block, which was fixed in formalin for a minimum of 48 hours and processed into paraffin wax block. One full thickness section (10 μm thick) was stained with hematoxylin and eosin (H&E) for histological assessment and micrographs at 40× and 100× magnifications were obtained.

## 3 RESULTS

### 3.1 Chorionic placental vasculature

**Figure 2:**
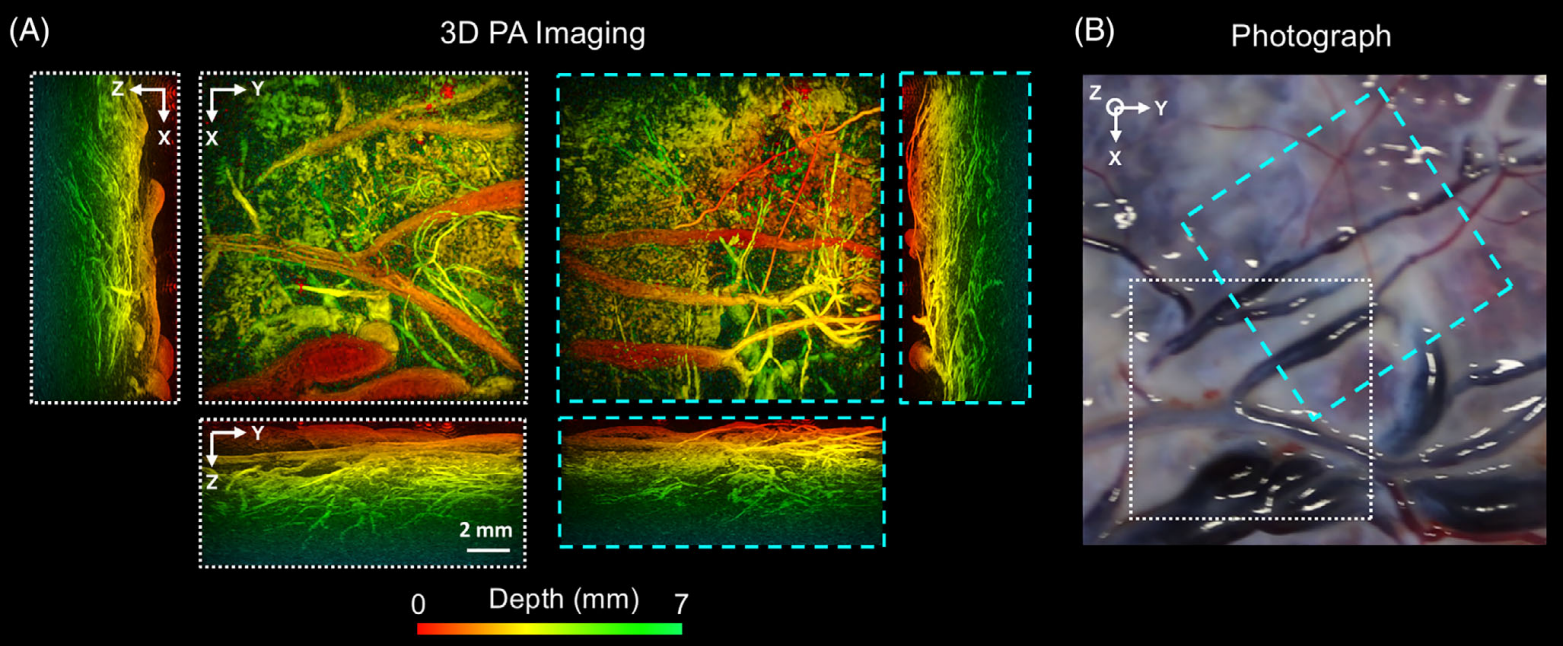
Photoacoustic (PA) images of the chorionic placental vasculature of a normal term placenta. The 3D PA images were obtained from two locations (white and cyan dashed squares) using the Fabry‐Pérot‐based planar sensor PA imaging system. They are displayed as maximum intensity projections of the reconstructed 3D image volume. The scale bar applies to all PA images. Superficial chorionic blood vessels are apparent in both *x*‐*y* PA images, A, and the corresponding photograph, B. Several subsurface structures that could be attributed to blood vessels are visible down to a depth of approximately 7 mm from the chorionic fetal surface of the placenta

Superficial chorionic placental vessels were clearly visualized using the F‐P based PA imaging system (Figure 2A). Good correspondence between the MIP PA images and the photograph (Figure 2B) was observed. Several subsurface objects, which could be attributed to blood vessels, were visible to a depth of approximately 7 mm from the chorionic fetal surface of the placenta. The smallest resolved subsurface vascular features were approximately 120 μm in diameter.

**Figure 3:**
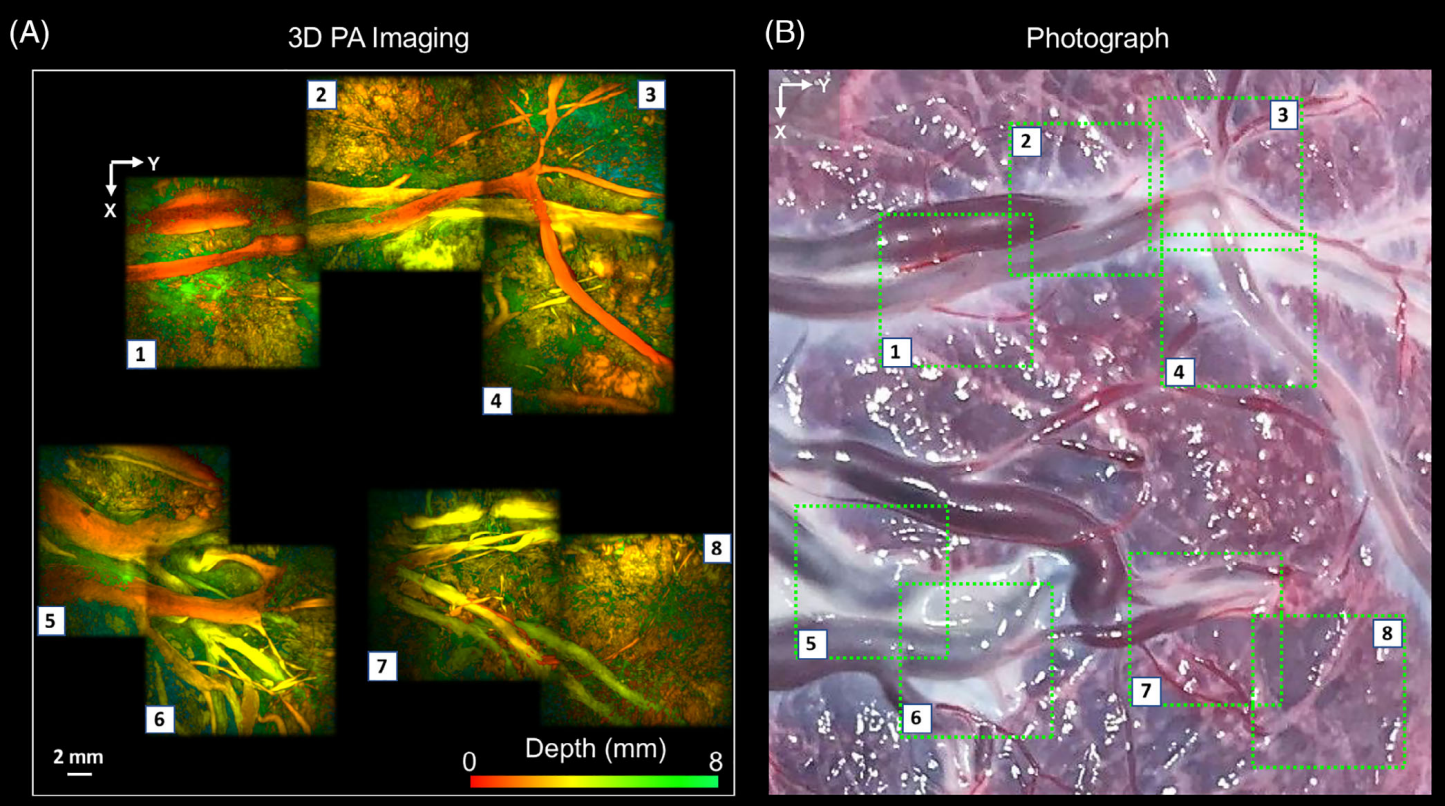
Photoacoustic (PA) images of the chorionic placental vasculature of an identical twin placenta treated for TTTS using laser photocoagulation of the placental vascular equator. The 3D PA images were obtained using a Fabry‐Pérot‐based PA scanner and are displayed as maximum intensity projections of the reconstructed 3D image volume. The images are arranged as a mosaic to capture a wider area of the placenta. Superficial blood vessels are clearly resolved, A, which correspond well to those apparent in the photograph, B

Manual mosaicking of 3D PA images, acquired from multiple locations of the chorionic placental vasculature of the TTTS‐treated placenta, allowed for a broader region of the placenta to be visualized (Figure 3). Superficial blood vessels were clearly resolved (Figure 3A), which corresponded well to those apparent in the photograph (Figure 3B). Subsurface vascular structures that were not visible in the photograph were apparent in the PA images to an approximate depth of 3 mm (Figure 3A; location 6).

**Figure 4:**
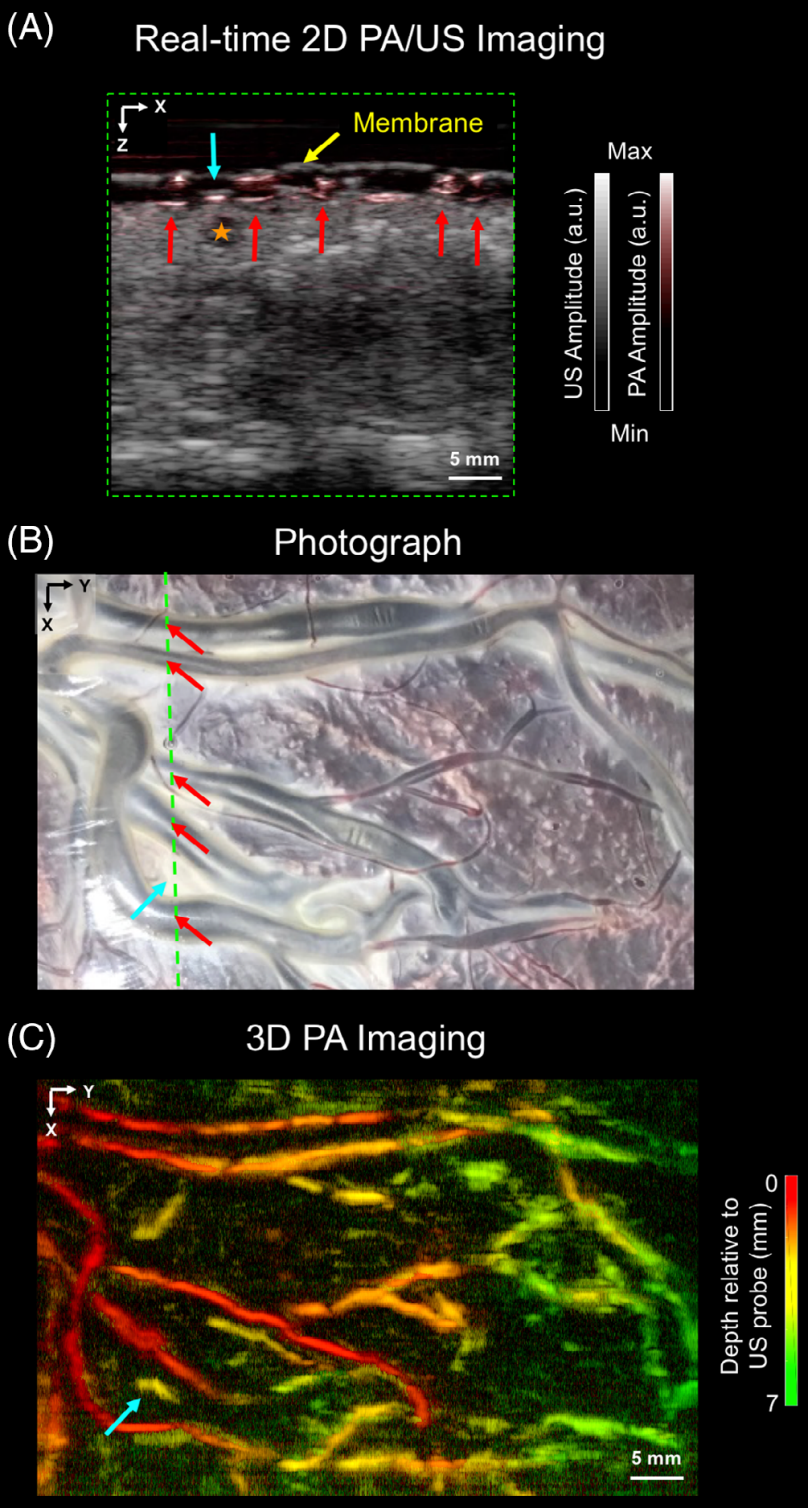
Wide‐field field photoacoustic (PA) and ultrasound (US) images of the chorionic placental vasculature in an untreated part of a TTTS photocoagulated placenta. The PA and US images were acquired using the clinical linear‐array US probe‐based PA imaging system. A, a 2D PA and a 2D US image, acquired from a region corresponding to the purple line in, B, are merged and displayed in a grayscale and a color‐scale, respectively. Several superficial blood vessels (red arrows) are visible in this 2D PA image but not clearly visible in the 2D US image; they correspond well to those apparent in the photograph, B. A prominent signal from the 2D PA image (solid blue arrow) was not apparent in the photograph, B. Additionally, a fluid‐filled cavity (orange star; 2D US image) was not visible in the 2D PA image. The placenta was covered with a cling film membrane (yellow arrow). The images are displayed on logarithmic scales. C, 3D PA image displayed as a maximum intensity projection of the reconstructed image volume, which was acquired by linear translation of the US probe. Superficial chorionic structures are clearly resolved, with good correspondence to the vessels apparent in the photograph, B

Wide‐field PA images and US images of the chorionic placental vasculature of the placenta treated for TTTS, which were acquired with the PA imaging system based on a clinical linear‐array US probe, are presented in Figure 4. Interleaved co‐registered PA and US images were displayed in real time (Figure 4A). With PA imaging, several superficial blood vessels were visualized (Figure 4A; red arrows), which corresponded well with those apparent in the photograph (Figure 4B). A prominent signal in the 2D PA image was not apparent in the photograph; it was also not readily visible in the US image. With US imaging, a hypoechoic region that likely corresponded to a fluid‐filled cavity (Figure 4A; star region) was not visible with PA imaging. This cavity potentially arose from water that leaked around the plastic membrane and may have entered the placenta from the maternal side. The 3D MIP PA image (Figure 4C) clearly revealed superficial branching vessels with good spatial correspondence with the photograph (Figure 4B).

### 3.2 Photocoagulated (scar) tissue

**Figure 5:**
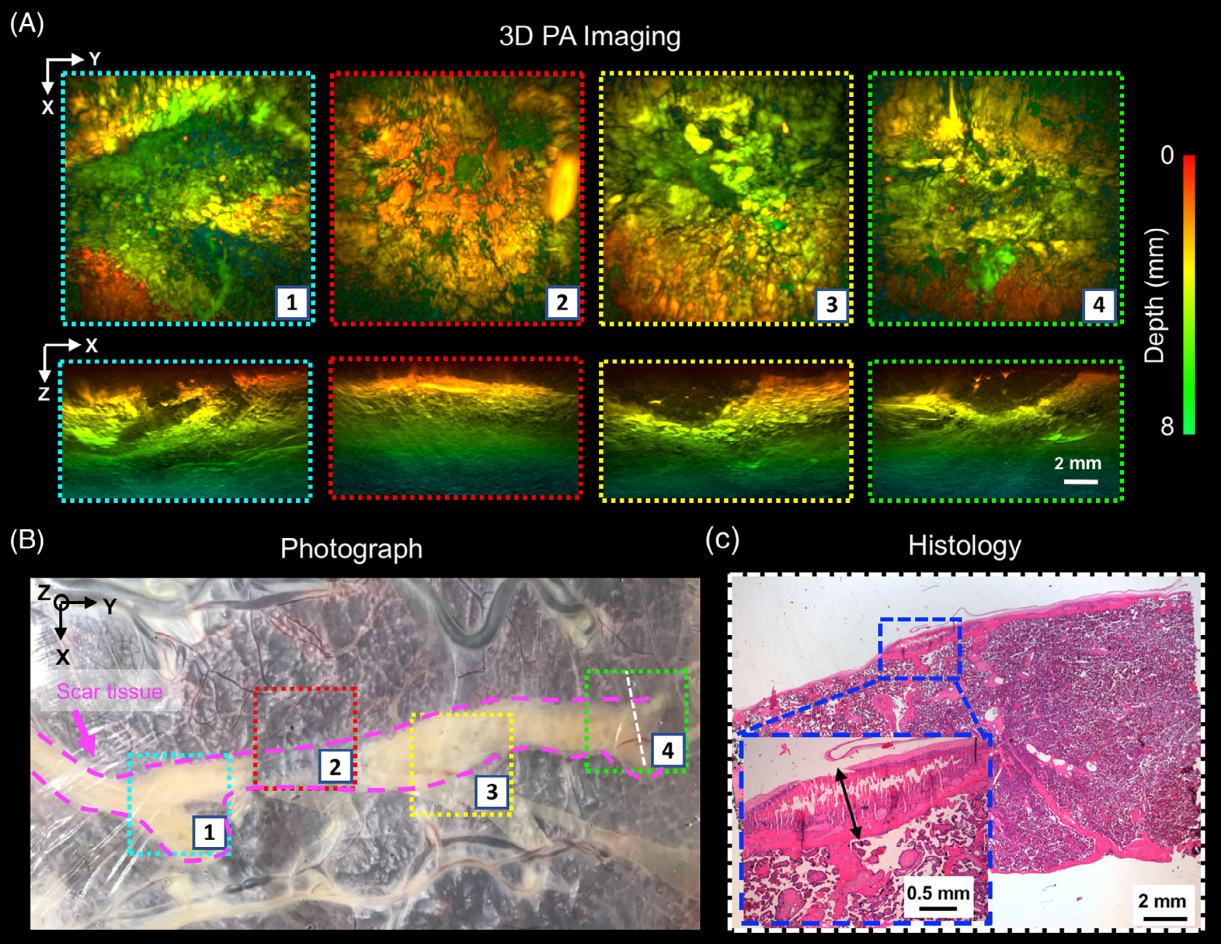
Photoacoustic (PA) images of the laser photocoagulated (scar) tissue of a TTTS‐treated placenta. A, The 3D PA images were obtained using a Fabry‐Pérot based PA scanner and are displayed on a logarithmic scale as maximum intensity projections (MIPs) of the reconstructed 3D image volume. The scar tissue is a weak absorber for excitation light and thereby provides negative contrast to the image. The photocoagulation depth is visible in the *x*‐*z* MIPs. The scale bar applies to all images and axes orientations. B, Corresponding photograph that includes the locations that were imaged in A. C, An H&E‐stained section was obtained from within imaging location 4 (dashed orange line). The photocoagulated tissue depth on histological examination is approximately 1 mm (double black arrow), which is consistent with that observed from the PA image acquired at the same location in A

PA images of photocoagulated tissue acquired with the F‐P based PA imaging system are presented in Figure 5. With PA imaging, negative contrast was obtained from scar tissues (Figure 5A). This decrease in signal with ablation with some previous studies 41, 42, where ablations led to reductions in PA signals. However, an increase in the Grüneisen coefficient with temperature can also result in an increase in the PA signal strength during real time ablation 43, 44. The photocoagulation depth across different locations in the scar was visible in the *x*‐*z* MIPs, and varied approximately from 2 to 4 mm. An H&E stained section obtained from one location (Figure 5B; location 4), confirmed the photocoagulation depth of approximately 1 mm (Figure 5C), which was found to be consistent with the corresponding PA image (Figure 5A; location 4).

**Figure 6:**
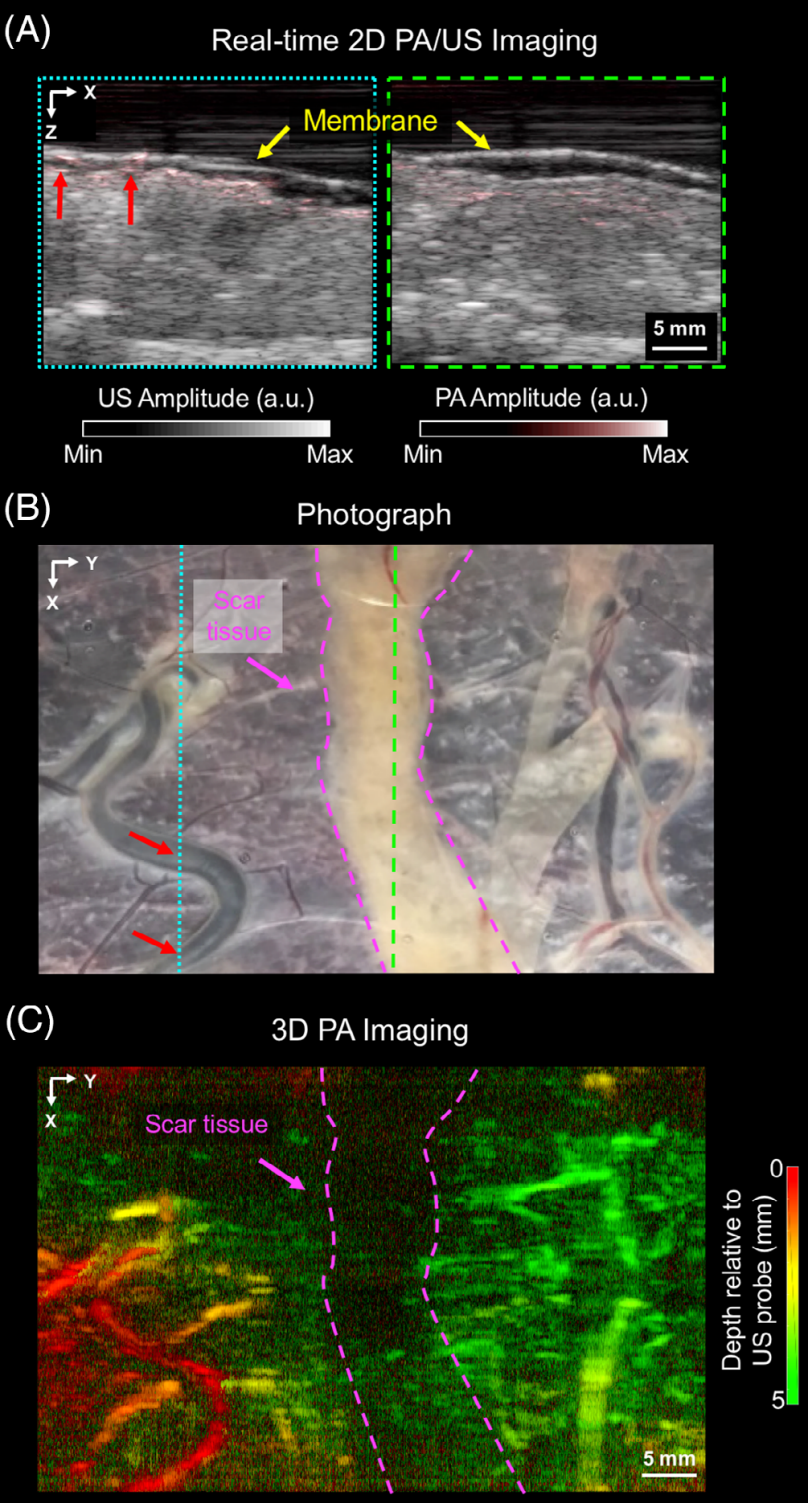
Wide‐field photoacoustic (PA) and ultrasound (US) images of tissue of a placenta that was treated for TTTS. The PA and US images were acquired with the PA/US imaging system based on a clinical linear array. A, Two frames of merged 2D PA and US images that were acquired from two locations: non‐treated tissue (dashed blue box in A corresponding to the dashed blue line in B) and scar tissue from photocoagulation treatment (dashed green lines). Superficial blood vessels (red arrows) are visible in the left PA image, which correspond well with those in the photograph, B. The placenta was covered with a cling film membrane (yellow arrow). The images are displayed on logarithmic scales. B, 3D PA image volume, which was acquired by linear translation of the US probe and displayed as a maximum intensity projection. Superficial chorionic structures are visualized, with good correspondence to the photograph, B

Wide‐field PA images of the scar tissue of a placenta treated for TTTS, which were acquired with the PA imaging system based on a clinical linear‐array US probe, are presented in Figure 6 In the absence of photocoagulation, prominent PA signal originated from superficial blood vessels (Figure 6A; red arrows), and there was good visual correspondence between the locations of those vessels and the photograph (Figure 6B). With photocoagulation, weak PA signals originating from locations beneath the scar tissue can be attributed to the relatively lower absorption of excitation light by the scar tissue as compared with nonphotocoagulated placental tissue. From the 3D MIP PA image (Figure 6C), superficial chorionic structures were visualized, and they corresponded well with the photograph (Figure 6B).

## 4 DISCUSSION

This study provides the first demonstration that volumetric PA imaging of the human placenta can generate detailed maps of surface and subsurface vasculature to a depth of approximately 7 mm. Fetoscopic photocoagulation, which was used to stop circulation across placental anastomoses in the vascular equator from identical twins during in utero TTTS treatment, was clearly visible. It manifested as an absence of signal resulting from weak absorption of PA excitation light in this region.

The two photoacoustic imaging systems used in this study provided complementary information. The F‐P based PA imaging system provided a much finer level of detail; the PA imaging system based on a clinical US probe allowed for rapid 2D imaging and concurrent B‐mode 2D US imaging. US imaging provided structural imaging that was absent in PA images and it facilitated interpretation of the PA images. As compared with previous 2D PA imaging studies in which excitation light was provided by an optical fiber 32, 33, the 3D PA images from both systems in this study provided a much more comprehensive visualization of placental vasculature.

Several questions remain unanswered. First, it is unclear what PA imaging depth range might be optimal for clinical use, although it is known that the depth range of anastomosing vessels in TTTS is quite shallow (a few mm) 8. Both systems used here had insufficient sensitivity to visualize vasculature at all depths in the placenta, and insufficient spatial resolution to visualize the smallest vessels. Imaging penetration depth depends on several factors, including the optical properties of the imaged tissue, the ultrasound bandwidth and sensitivity of the F‐P sensor, and the physical dimensions of the F‐P sensor. The human placenta is a highly vascularized tissue and since it was imaged ex vivo, contributions of deoxy‐hemoglobin are likely dominant at 760 nm compared to oxy‐hemoglobin, which is more abundant in vivo. Therefore, penetration depths are likely to be higher at 760 nm when imaging is performed in vivo. Typically, lower frequency systems allow for imaging at greater depths. The penetration depths achieved in this study reflect high‐frequency (high‐resolution) imaging and are broadly consistent with previous studies 26, 34, 35, 36. Next‐generation systems could also include ultrasound receivers with ultra‐high sensitivities 45 and coded excitation sequences 46. Second, it is unclear what lateral field of view and spatial resolution may be required for clinical use. If needed, the lateral field of view could be increased using actuators such as those based on McKibben muscles 47, and the use of haptic guidance 48. Third, given that volumetric imaging can be time consuming, it remains to be seen whether it would be efficient to direct it at specific locations on the placenta. There is variability in the reported distribution of residual anastomoses across the surface of placentas, with reports of a higher density at the placental margin 49 and a relatively uniform spread along the vascular equator 12. In current clinical practice, photocoagulation can be particularly challenging when there are proximate cord insertions (distance <5 cm between the two umbilical cord insertions) 50 and when there are anastomoses away from the placental vascular equator, particularly those involving small vessels (0.4 ± 0.5 mm) at the edge of the placenta 12. The photocoagulation depth and the vasculature are likely to change during pregnancy. A study by Branisteanu‐Dumitrascu et al 51 measured the in vivo impact of photocoagulation and found that the local effects of the photocoagulation did not significantly change over time; however, there were significant collateral and peripheric tissue changes. Additionally, secondary changes and collateral damage are limited due to the placental perfusion and healing as the maternal circulation keeps tissue components alive.

For clinical translation of PA imaging to guide TTTS treatment in utero, it will be necessary to develop miniature probes with sufficiently small lateral dimensions that allow for excitation light delivery through the working channel of a fetoscope (typically less than 2 mm in diameter 52) and with real‐time imaging 32. Intraoperative guidance could be achieved with the development of miniature, forward‐viewing PA and US endoscopic probes 53, 54, 55, 56, 57, 58, 59 in concert with recent developments in real‐time imaging capabilities enabled by parallel data acquisition 36 and compressed sensing 60. Multispectral PA imaging, with multiple excitation wavelengths, could be employed to obtain information about hemoglobin oxygen saturation in the vasculature 16, 17, 18 and potentially to provide insights about the pathophysiology of TTTS and fetal growth restriction. Additional interventional procedures that could potentially benefit from the combination of PA imaging and ultrasound include ablation monitoring during laser or radiofrequency therapy of lesions such as those in the heart 41, 44 and in the liver 42.

In conclusion, PA imaging in 3D appears to be promising for visualizing human placental vasculature in healthy and TTTS‐treated placentas. Improving the detection of residual anastomosing vessels at the time of photocoagulation therapy for TTTS in utero is likely to improve patient outcomes by achieving complete dichorionization. By visualizing superficial and subsurface chorionic placental vasculature, interventional PA imaging could be valuable for minimally invasive fetal therapies.
